# Genome analysis of *Pseudoalteromonas flavipulchra* JG1 reveals various survival advantages in marine environment

**DOI:** 10.1186/1471-2164-14-707

**Published:** 2013-10-16

**Authors:** Min Yu, Kaihao Tang, Jiwen Liu, Xiaochong Shi, Tobias AM Gulder, Xiao-Hua Zhang

**Affiliations:** 1 College of Marine Life Sciences, Ocean University of China, Qingdao 266003, China; 2 Kekulé-Institute of Organic Chemistry and Biochemistry, University of Bonn, Gerhard-Domagk-Straße 1, Bonn 53121, Germany; 3 Mailing address: College of Marine Life Sciences, Ocean University of China, 5 Yushan Road, Qingdao 266003, China

**Keywords:** *Pseudoalteromonas flavipulchra*, Genome analysis, Antibacterial metabolites, Quorum quenching, Survival advantages

## Abstract

**Background:**

Competition between bacteria for habitat and resources is very common in the natural environment and is considered to be a selective force for survival. Many strains of the genus *Pseudoalteromonas* were confirmed to produce bioactive compounds that provide those advantages over their competitors. In our previous study, *P. flavipulchra* JG1 was found to synthesize a *Pseudoalteromonas flavipulchra* antibacterial Protein (PfaP) with L-amino acid oxidase activity and five small chemical compounds, which were the main competitive agents of the strain. In addition, the genome of this bacterium has been previously sequenced as Whole Genome Shotgun project (PMID: 22740664). In this study, more extensive genomic analysis was performed to identify specific genes or gene clusters which related to its competitive feature, and further experiments were carried out to confirm the physiological roles of these genes when competing with other microorganisms in marine environment.

**Results:**

The antibacterial protein PfaP may also participate in the biosynthesis of 6-bromoindolyl-3-acetic acid, indicating a synergistic effect between the antibacterial macromolecule and small molecules. Chitinases and quorum quenching enzymes present in *P. flavipulchra*, which coincide with great chitinase and acyl homoserine lactones degrading activities of strain JG1, suggest other potential mechanisms contribute to antibacterial/antifungal activities. Moreover, movability and rapid response mechanisms to phosphorus starvation and other stresses, such as antibiotic, oxidative and heavy metal stress, enable JG1 to adapt to deleterious, fluctuating and oligotrophic marine environments.

**Conclusions:**

The genome of *P. flavipulchra* JG1 exhibits significant genetic advantages against other microorganisms, encoding antimicrobial agents as well as abilities to adapt to various adverse environments. Genes involved in synthesis of various antimicrobial substances enriches the antagonistic mechanisms of *P. flavipulchra* JG1 and affords several admissible biocontrol procedures in aquaculture. Furthermore, JG1 also evolves a range of mechanisms adapting the adverse marine environment or multidrug rearing conditions. The analysis of the genome of *P. flavipulchra* JG1 provides a better understanding of its competitive properties and also an extensive application prospect.

## Background

The genus *Pseudoalteromonas* was established in 1995 and was found to be ubiquitous in the marine environment [1]. Numerous *Pseudoalteromonas* strains were isolated from the polar region, inshore waters or surfaces of marine organism, and were shown to synthesize a range of bioactive molecules [2-4]. The production of molecules that are active against a variety of target organisms appears to be an important characteristic for this genus and may greatly benefit *Pseudoalteromonas* cells in their competition for nutrients or colonization of surfaces [5]. *P. tunicata* D2, as a model organism to study, was demonstrated to synthesize a range of inhibitory substances, including a toxic antibiotic protein and two pigments [6,7]. Analysis of its complete genome sequence revealed that several genes and gene clusters were involved in the production of inhibitory compounds that were associated with its successful persistence and competition on marine surfaces [8].

*P. flavipulchra* JG1 was isolated from rearing water of healthy turbot (*Scophthalmus maximus*) in Qingdao, China. Strain JG1 is capable of adapting to the oligotrophic marine environment and reveals various advantageous survival abilities among competitive species. It was demonstrated to exhibit inhibitory activity against many *Vibrio*, *Aeromonas* and *Bacillus* strains and was nontoxic to zebra fish and mantis shrimp [9]. Furthermore, Strain JG1 was shown to synthesize the putative L-amino acid oxidase named *Pseudoalteromonas flavipulchra* antibacterial Protein (PfaP) and 5 small molecular compounds with antibacterial activity. These compounds were identified as *p*-hydroxybenzoic acid, *trans*-cinnamic acid, 6-bromoindolyl-3-acetic acid, *N*-hydroxybenzoisoxazolone and 2′-deoxyadenosine. All of these metabolites have been observed to exhibit antibacterial activities against several pathogens, including *V. anguillarum*, *V. harveyi*, *Photobacterium damselae* subsp. *damselae* and *A. hydrophila*[10]. The inhibitory properties of *P. flavipulchra* JG1 against fish pathogens indicate that the strain or its products could be utilized as biocontrol agent(s) in aquaculture.

To obtain a better understanding of the genetic potential of *P. flavipulchra* JG1 as a biocontrol organism, we have sequenced and analyzed its genome and compared it to the genomic data of closely related strains publicly available. We have found that the *P. flavipulchra* genome contains several genes and gene clusters that might be involved in the production of inhibitory compounds against pathogens and competitors in the marine environment. The analysis of *P. flavipulchra* genome also verifies excellent capabilities of this strain to adapt to environmental changes and challenges.

## Results and discussion

### Genome features and comparison with other *Pseudoalteromonas* genomes

**Figure 1:**
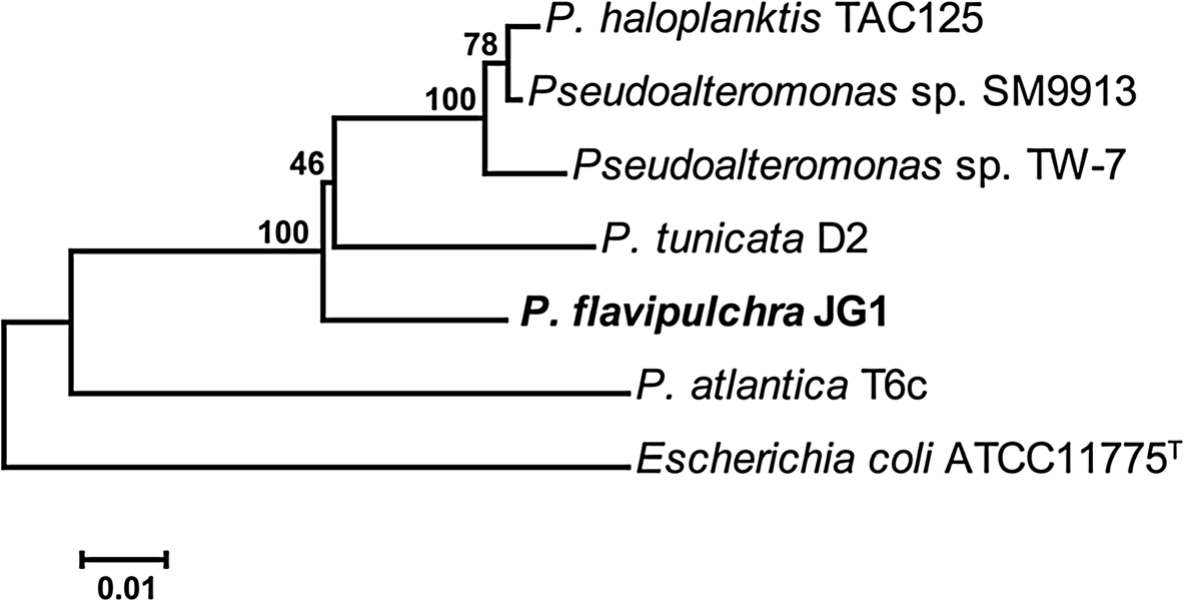
**Phylogenetic tree based on 16S rDNA of JG1 and *Pseudoalteromonas *strains compared in the study.** Tree was generated by Neighbor-Joining method with 1000 bootstrap replications. The sequence alignment and phylogenetic calculations were performed with MEGA 5.

The *P. flavipulchra* JG1 genome is composed of 5 565 361 bp and the calculated G + C content is 43.23%. A total of 4 913 open reading frames (ORFs) are identified within the *P. flavipulchra* JG1 genome (Table 1). Among the predicted genes, 1 985 (40.4%) are not found in COG categories, and 1 725 (35.1%), 712 (14.5%), 1 863 (37.9%) and 629 (12.8%) genes are not applicable within the KEGG, NR, SwissProt and TrEMBL databases, respectively [11]. JG1 contains 180 tandem repeat regions, 143 transposons, and 5 ISs (Insert sequences) that account for about 1.1% of the genome. The *P. flavipulchra* JG1 genome is larger than that of the five other *Pseudoalteromonas* strains whose genomic sequences have been published in the IMG database: *P. tunicata* D2 (Gi05080), *P. haloplanktis* TAC125 (Gc00289), *P. atlantica* T6c (Gc00395) and two *Pseudoalteromonas* strains TW-7 (Gi01432) and SM9913 (Gc01563). D2 is an anti-biofouling bacterium, both T6c and TW-7 are mesophile bacteria, and TAC125 and SM9913 are psychrotolerant adapting to cold aqueous and deep-sea sediment environment, respectively. General features of these genomes were given in Table 1 and their phylogenetic relationship based on the 16S rRNA gene (Figure 1) indicated that *P. flavipulchra* JG1 formed a cluster with *P. tunicata* D2. Comparing with other sequenced *Pseudoalteromonas* strains, *P. tunicata* D2 [12] also exhibited great inhibitory activities against several specific organisms as in *P. flavipulchra* JG1 [10], indicating that *P. flavipulchra* was functionally closely related to *P. tunicata* D2.

**Table 1:** **General features of JG1 and other *Pseudoalteromonas *genomes**

	***P. flavipulchra *JG1**	***P. tunicata *D2**	***P. atlantica *T6c**	***P. haloplanktis *TAC125**	***Pseudoalteromonas *sp. TW-7**	***Pseudoalteromonas *sp. SM9913**
Genome size	5 505 361	4 982 425	5 187 005	3 850 272	4 104 952	4 037 671
CDS number	4913	4505	4313	3487	3783	3712
CDS length	4 828 917	4 421 622	4 507 935	3 404 858	3 673 686	3 569 827
G + C percentage	43%	40%	45%	40%	40%	40%
RNA number	158	133	92	147	94	87
CDS assigned to COG	3314	3096	3279	2639	2797	2860
COG Cluster number	1801	1791	1846	1731	1778	1768

**Figure 2:**
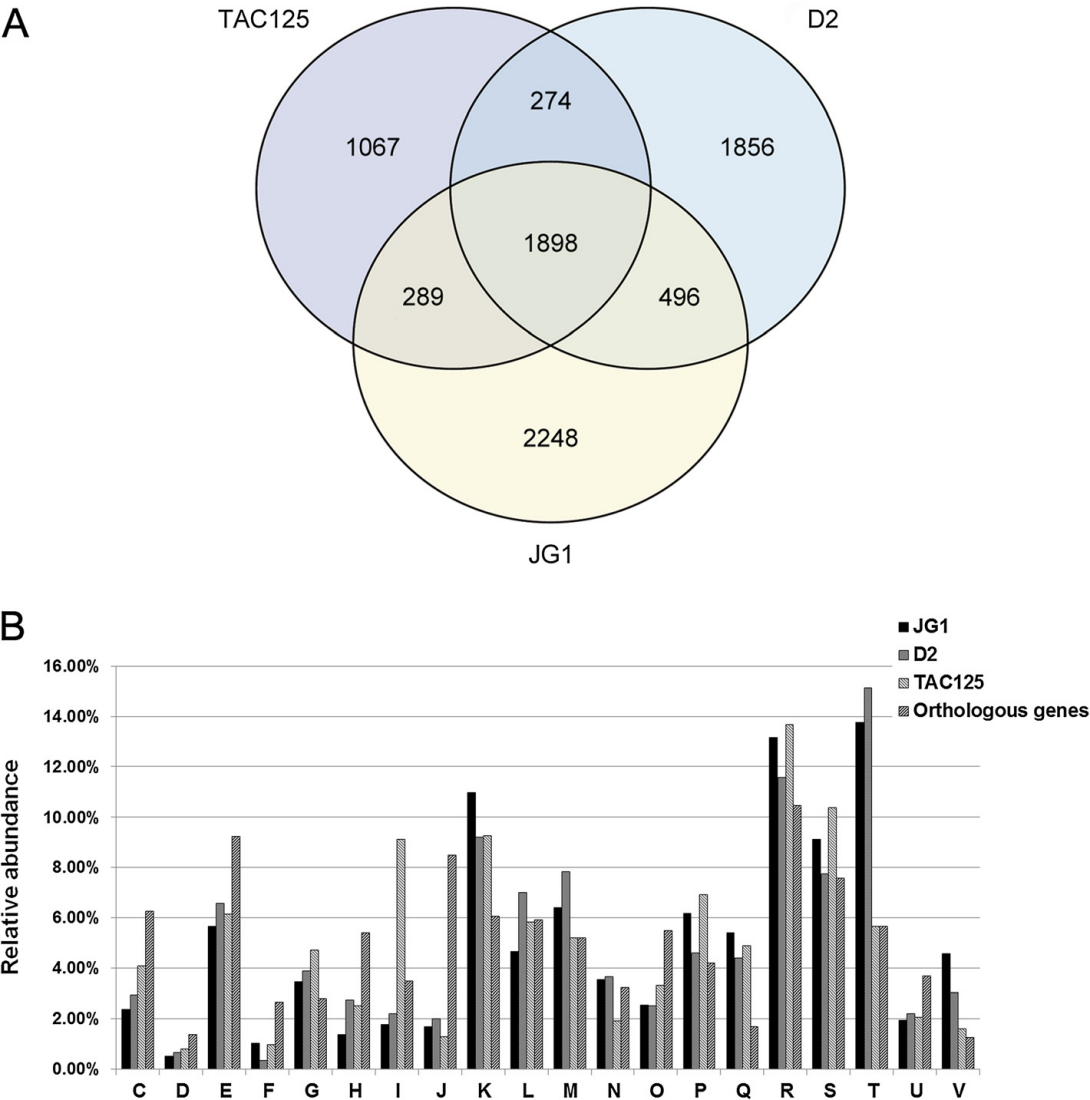
**Comparison of the gene content of *P. flavipulchra *JG1, *P. tunicata *D2 and *P. haloplanktis *TAC125.** Putative orthologous genes are defined as reciprocal best hit proteins with a minimum 40% identity and 70% of the length of the query protein, calculated by the BLAST algorithm. **(A)** Venn diagram of the orthologous and specific genes in each strain. **(B)** Relative abundance compared to all COG categories of the orthologous and specific genes in each strain. COG functional categories are described as follows: C, energy production and conversion; D, cell cycle control, cell division, chromosome partitioning; E, amino acid transport and metabolism; F, nucleotide transport and metabolism; G, carbohydrate transport and metabolism; H, coenzyme transport and metabolism; I, lipid transport and metabolism; J, translation, ribosomal structure and biogenesis; K, transcription; L, replication, recombination and repair; M, cell wall/membrane/envelope biogenesis; N, cell motility; O, posttranslational modification, protein turnover, chaperones; P, inorganic ion transport and metabolism; Q, secondary metabolites biosynthesis, transport and catabolism; R, general function prediction only; S, function unknown; T, signal transduction mechanisms; U, intracellular trafficking, secretion, and vesicular transport; V, defense mechanisms.

*P. flavipulchra* JG1, *P. tunicata* D2 and *P. haloplanktis* TAC125 share 1898 orthologous genes, accounting for 38.63%, 42.14% and 54.47% of all genes of JG1, D2 and TAC125, respectively. In addition, JG1 contains more specific genes (2248, 45.76%) than D2 (1856, 41.21%) and TAC125 (1067, 30.63%), and there are more common genes within JG1 and D2 (Figure 2A). We have also conducted a COG-based analysis among the specific and orthologous genes of these three strains. As shown in Figure 2B, a larger proportion of JG1 and D2 specific genes belong to signal transduction mechanisms (T), while the genome of TAC125 harbors more specific genes belonging to lipid transport and metabolism (I). Genes involved in signal transduction were considered to allow *P. tunicata* D2 to generate phenotypic variation and provide the capacity of niche adaptation [8], and genes assigned to the same COG category might also play a similar role in *P. flavipulchra* JG1 in that both D2 and JG1 exhibited competitive activities in their surrounding environments. Contrasting, in order to adapt cold condition, TAC125 needs more genes involved in lipid transport and metabolism such as genes coding for lipid desaturase, which could increase membrane fluidity at low temperature [13]. According to the genomic comparison results, JG1 and D2 share more functional similarities than to TAC125.

Significantly, *P. flavipulchra* contains the highest proportion of specific genes belonging to the COG categories defense mechanisms (V) and secondary metabolites biosynthesis, transport and catabolism (Q), which account for 4.56% and 5.40%, respectively, among other bacteria. (Additional file 1: Figure S1). This mainly attributes to more genes involved in ABC-type antimicrobial peptide transport system (COG0577, COG1136), beta-lactamase class C and other penicillin binding proteins (COG1680), cation/multidrug efflux pump (COG0841), ABC-type siderophore export system (COG 4615) and those related to the synthesis of potential bioactive compounds, such as nonribosomal peptide synthetase (NRPS) modules (COG1020) and polyketide synthases (COG 3321). The abundance of genes involved in expression and transport of potential primary and secondary metabolites and defense compounds are consistent with the capability of *P. flavipulchra* to produce various antimicrobial compounds [10] and generate survival advantages in marine environments [8].

### Biosynthesis of antimicrobial metabolites

*P. flavipulchra* JG1 was demonstrated to synthesize the antibacterial protein PfaP and 5 known small molecular compounds with antibacterial activity against *V. anguillarum*[10].

**Figure 3:**
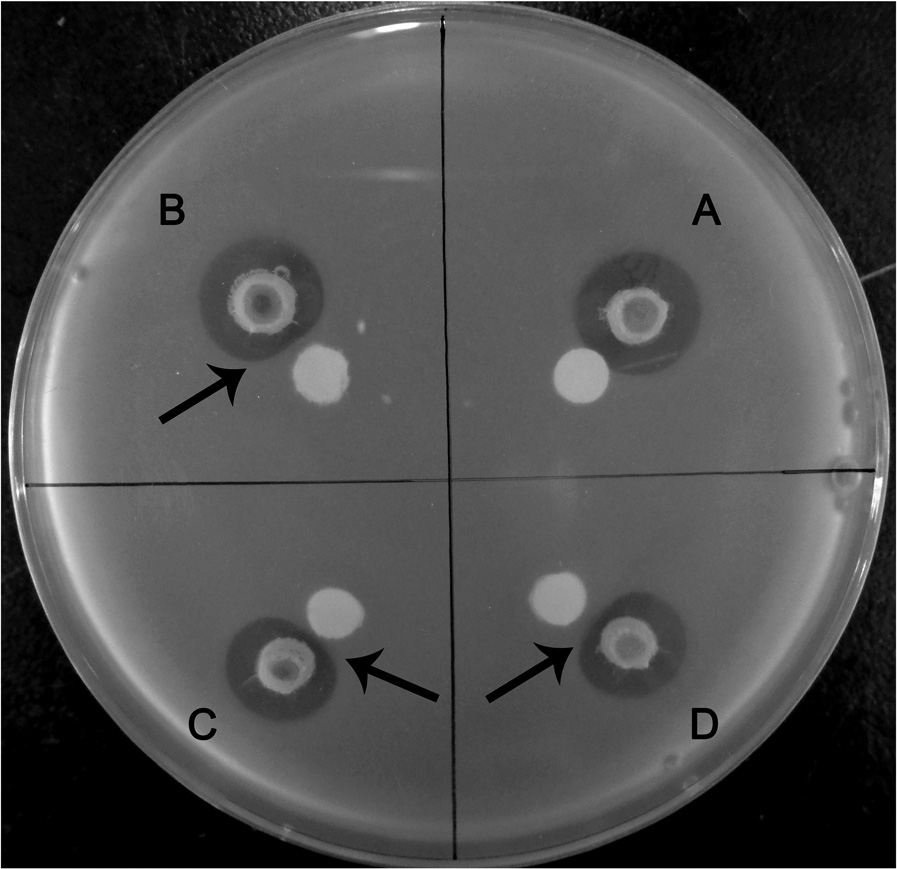
**Catalase inhibition of the antibacterial effect of extracellular proteins against *Vibrio anguillarum*. A**, disk with distilled water; **B**, disk with 0.1 mg of catalase; **C**, disk with 0.2 mg of catalase; **D**, disk with 0.5 mg of catalase.

The antibacterial protein PfaP is a homologue of L-amino acid oxidase with high similarity to the L-lysine oxidase AlpP of *P. tunicata* D2 (GenBank AAP73876.1) [6] and marinocine antimicrobial protein of *Marinomonas mediterranea* MMB-1 (GenBank AAY33849.1) [14]. The sequence identities of protein PfaP and these two antimicrobial proteins were 58% and 54%, with coverages of 95% and 92%, respectively. PfaP is a secreted protein that might catalyze the oxidative deamination of specific amino acids to the respective α-keto acids with concomitant release of ammonium and hydrogen peroxide [15]. The antibacterial activity of extracellular proteins of JG1 could be abolished in the presence of catalase, suggesting that the inhibitory effect was directly mediated by the action of hydrogen peroxide (Figure 3). The gene located downstream of *pfaP* (FaGL1049) codes a catalase (FaGL1053) which responds to oxidative stress and causes decomposition of hydrogen peroxide. The catalase activity might thus immediately protect JG1 from the damage of the hydrogen peroxide generated by PfaP. We also identified 3 further genes encoding catalases and several genes capable of hydrogen peroxide degradation in the genome. Thus, JG1 produces the antimicrobial protein PfaP and simultaneously possesses several genes related to potential self-protection mechanisms.

The compounds *p*-hydroxybenzoic acid, *trans*-cinnamic acid, and 6-bromoindolyl-3-acetic acid isolated from *P. flavipulchra* JG1 were demonstrated to inhibit the growth of several pathogens [10]. We now identified several genes most likely involved in the biosynthesis of these metabolites.

Compound *p*-hydroxybenzoic acid and its derivatives are widely applied as food preservatives and stabilizers (antioxidants). As an organic acid, it has also been shown to inhibit the growth of ethanologenic *Escherichia coli* LY01 and ethanol synthesis [16]. The compound is probably derived from chorismate by action of a chorismate lyase, resembling the first step of ubiquinone biosynthesis. *p*-hydroxybenzoic acid is an important precursor of ubiquinones and *P. flavipulchra* harbors the *ubiCA* gene cluster (FaGL1386 and FaGL1387) responsible for the conversion of chorismate to *p*-hydroxybenzoic and then to 3-octaprenyl-4-hydroxybenzoate, which are the first two steps of the biosynthesis of ubiquinone [17]. Ubiquinone is not only an essential component of the aerobic respiratory chain, but functions in the reduced form (ubiquinol) as an antioxidant, significantly reducing oxidative stress, as for example generated by hydrogen peroxide [18]. We also identified all other genes in JG1 that are necessary for ubiquinone production.

For the biosynthesis of *trans*-cinnamic acid, two biosynthetic pathways can be taken into consideration: it can be derived from tyrosine by tyrosine ammonia lyase (TAL) directly [19], or cinnamic acid could get hydroxylated after its formation by phenylalanine ammonia lyase (PAL) [20]. Lyase-catalyzed reductive deamination releases ammonia thus forming the *trans* double bond found in the compound. This reaction sequence has been confirmed in *Streptomyces maritimus*[21] and *Rhodobacter capsulatus*[22]. However, in the genomic data of *P. flavipulchra* JG1 only two homologs of TAL (FaGL3502 and FaGL4409) can be identified. Therefore, this compound is most probably derived from tyrosine in JG1. *trans*-cinnamic acid is often found in plants. It has been used to augment the activity of various antibiotics against *Mycobacterium avium*[23] and exhibited synergistic effects with several anti-tuberculosis drugs active against *M. tuberculosis*[24]. In contrast to its wide occurrence in plants, *trans*-cinnamic acid is not very common in bacteria and only few reports on the biochemical characterization of TAL have been published [19].

**Figure 4:**
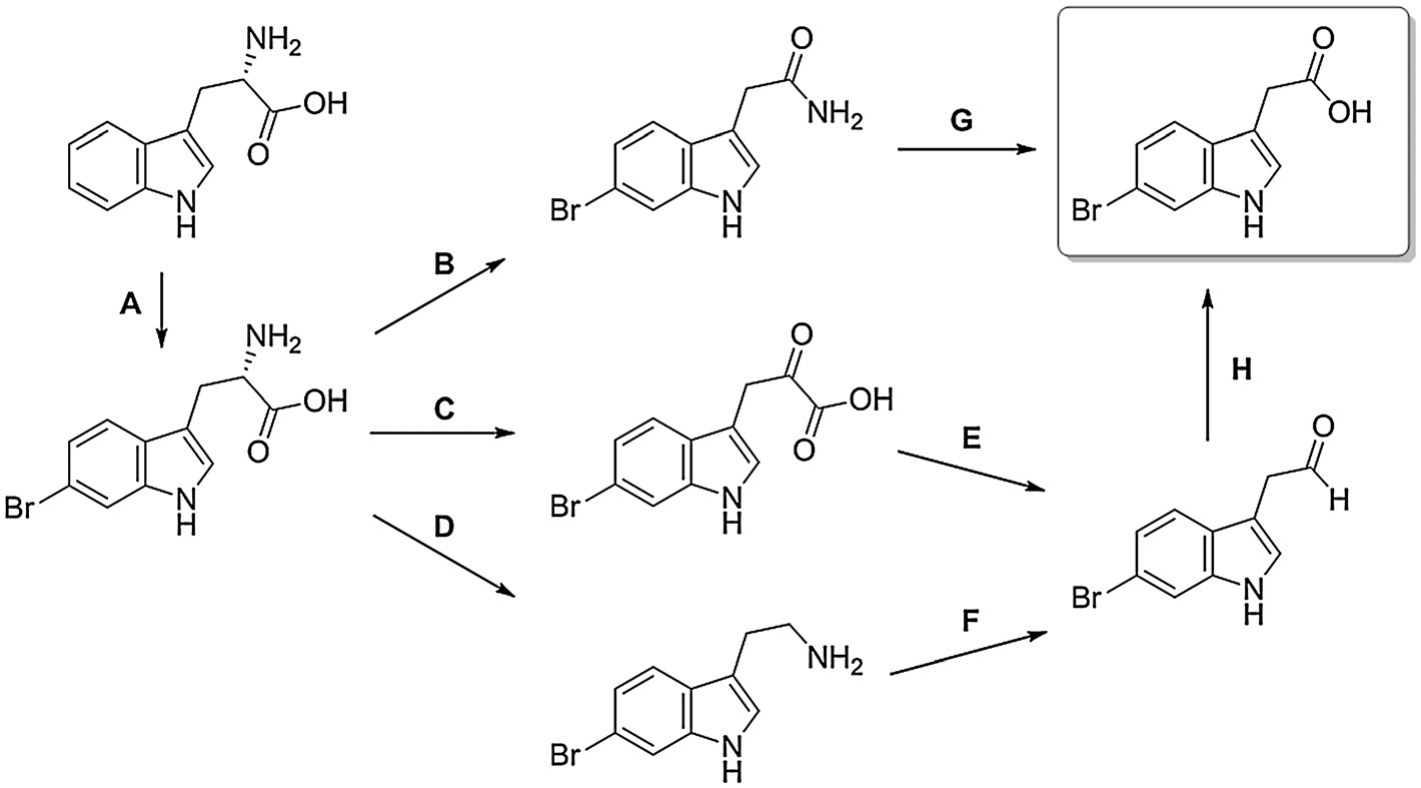
**The biosynthesis pathways of 6-bromoindolyl-3-acetic acid in JG1. A**, tryptophan halogenase; **B**, amino acid monooxygenase; **C**, L-amino acid oxidase; **D**, tryptophan decarboxylase; **E**, indole-3-pyruvate decarboxylase; **F**, monoamine oxidase; **G**, indoleacetamide hydrolase; **H**, aldehyde dehydrogenase.

Compound 6-bromoindolyl-3-acetic acid was shown to exhibit the strongest antibacterial activity among the small molecular compounds so far isolated from *P. flavipulchra* JG1. The metabolite can be formed by halogenation of tryptophan followed by oxidative deamination and decarboxylation. The indole-3-acetic acid (IAA) portion can be formed in bacteria following several IAA biosynthesis pathways, of which a single bacterial strain sometimes contains more than one [25]. IAA can be converted from indole-3-acetamide and indole-3-acetaldehyde via the indole-3-pyruvic acid pathway or the tryptamine pathway in JG1. The key enzymes involved in both of these pathways are present in the genome of JG1, including amino acid monooxygenase, indoleacetamide hydrolase, indole-3-pyruvate decarboxylase and monoamine oxidase. However, the tryptophan aminotransferase which transforms tryptophan into indole-3-pyruvate has not been detected in JG1. Alternatively, the reaction could be mediated by an L-amino acid oxidase and the PfaP protein might be a candidate. In addition, genes encoding tryptophan halogenases are found in the genome of JG1, one of which (FaGL1050) is located just downstream of *pfaP*. A tryptophan halogenase would catalyze bromination of tryptophan, which subsequently would be converted into 6-bromoindolyl-3-acetic acid via one of the above mentioned biosynthetic pathways (Figure 4). The PfaP protein, which is considered as a multifunctional protein, might be the most important antibacterial factor of *P. flavipulchra* thus far identified, as it also might play a role in the biosynthesis of 6-bromoindolyl-3-acetic acid.

To assess the potential of *P. flavipulchra* JG1 to produce secondary metabolites that have not yet been isolated and chemically characterized, we analyzed its genome using antiSMASH [26]. This allowed for the identification of four different bacteriocin-type gene clusters, lantipeptide biosynthesis genes, four type I polyketide/non-ribosomal peptide (PKS/NRPS) hybrid clusters, three NRPS gene clusters (Additional file 2: Table S1), as well as a high number of further NRPS-related peptidyl-carrier proteins (12), condensation (11), adenylation (12), epimerization (3), and thioesterase (2) domains distributed in the smaller scaffolds derived from our sequencing efforts. This strain thus appears to harbor tremendous potential to produce a large variety of peptide-based secondary metabolites. It is interesting to note that in terms of polyketide biosynthesis only type I PKS genes have been identified, all of these being part of larger NRPS biosynthetic loci. Among the biosynthetic gene clusters predicted by antiSMASH analysis, we identified a set of genes that can be correlated to polycyclic tetramic acid containing macrolactams (PTMs). This class of secondary metabolite gene clusters was recently found to be widespread among bacterial species and encodes the assembly of structurally complex, polycyclic secondary metabolites with a broad range of biological activities [27]. Given the apparent genetic potential of *P. flavipulchra* JG1 to produce complex bioactive natural products, further studies to chemically unlock its secondary metabolite profile seem to be extremely promising.

Besides these secondary metabolites potential, *P. flavipulchra* JG1 was shown great chitinase activity on chitin agar. Chitinase can catalyze the hydrolysis of β-1, 4 glycosidic bonds linking the *N*-acetylglucosamine subunits of chitin in a variety of organisms, and the enzyme has been found in all sorts of organisms including bacteria, fungi, plants and animals [28,29]. Moreover, chitinase can be classified into two families as glycoside hydrolases (GH) 18 and 19 based on the amino acid sequence similarity [30,31]. *P. flavipulchra* JG1 harbored 5 genes (faGL1724, faGL2271, faGL2273, faGL2526 and faGL4793) coding for this enzyme, four of which belong to GH18. In the genomes of *P. tunicata* D2 and *Pseudoalteromonas* strains SM9913, by comparison, only two genes are annotated as chitinases, while no genes encoding this enzyme are found in *P. haloplanktis* TAC125 and *P. atlantica* T6c. Notably, chitinase has been confirmed to show antifungal activities in *B. cereus*[32] and *Streptomyces* sp. MG3 [28] as chitin is a component of the cell walls of fungi. Two extracellular chitinases purified from *Pseudomonas aeruginosa* K-187 also showed lysozyme activities against both gram-positive and gram-negative bacteria [33]. The chitinases identified in JG1 can thus be considered as antibacterial/antifungal proteins with a different antagonizing mechanism.

### Quorum sensing and quorum quenching

Quorum sensing is a process that has been developed by bacteria to allow communication with each other and to facilitate monitoring of their cell density by measuring the concentration of small secreted signal molecules, such as acyl homoserine lactone (AHL) [34]. Diverse functions of bacteria are regulated via quorum sensing systems, including antibiotic biosynthesis, motility patterns and expression of many virulence-related genes in some animal and plant pathogens [35]. The quorum sensing regulatory system QseB/QseC detected in the genome of JG1 is organized in an operon with *qseB* encoding the response regulator and *qseC* the sensor histidine kinase. QseB/QseC belongs to two-component signal transduction systems which enable bacteria to sense, respond, and adapt to changes in their environment or in their intracellular state [36]. However, the major genes encoding autoinducers responsible for quorum sensing have not been detected in JG1. The system may thus only regulate genes encoding the expression and assembly of flagella, motility and chemotaxis in JG1, as a protein responsible for biogenesis of type IV pilus is just downstream to the QseB/QseC system.

Quorum quenching (QQ) describes all processes that interfere with quorum sensing [37]. QQ enzymes, including AHL lactonase, acylase, oxidase and reductase, have been discovered in many bacterial genuses, such as *Bacillus*, *Rhodococcus*, *Pseudomonas* and *Ralstonia*[38]. Extra-and intra-cellular products of strain JG1 were shown to degrade long-chain AHLs, such as C10-HSL, C12-HSL, C14-HSL, 3-oxo-C12-HSL and 3-oxo-C14-HSL, but not short-chain AHLs (shorter than 10 carbons in the acyl chain), and the enzymatic activity could not be reversed by acidification. This suggested that there might be genes encoding AHL acylases in the genome of strain JG1. Two genes of JG1 (FaGL1422 and FaGL2554) encoding penicillin acylase show high homology with PvdQ [39] and QuiP [40], which in *Pseudomonas aeruginosa* PAO1 were demonstrated to degrade long chain AHLs. Moreover, QuiP could utilize long chain AHLs as sole sources of carbon and energy. The catalytically active serine residue of N-terminal nucleophilic hydrolases, as found in the functionally verified AHL acylases PvdQ and QuiP, is present in both homologs found in JG1. These potential AHL acylases therefore equip strain JG1 with another mechanism to reduce the detrimental effects of pathogens and prevent or limit the impact of bacterial diseases in rearing animals.

### Acquisition of phosphorus

Efficient uptake of phosphorus is important for marine microorganisms due to the low phosphorus level in the marine environment. The phosphate input in the metabolism of *P. flavipulchra* JG1 is controlled by the counterparts of PhoB/PhoR and PhoU [41] as well as several phosphate transport systems (PstABC and PstS) [42] and inorganic phosphate (Pi) transport systems (Pit) [43]. The Pst system, which is derepressed under conditions of Pi starvation, also regulates synthesis of alkaline phosphatase, a periplasmic protein produced in greatest quantity during Pi starvation [44]. Homologs to the two component regulatory system PhoR/PhoB found in JG1 are responsible to control phosphate starvation and the system may also serve as a general transduction system for the expression of genes involved in secondary metabolism. Significantly, Pi starvation may stimulate bacteria to produce various secondary metabolites. For example, antibiotics biosynthesis in *Streptomyces lividans* is negatively regulated by phosphate via the PhoR/PhoB system [45]. JG1 might therefore up-regulate the production of defensive primary and secondary metabolites in the oligotrophic marine environment, thereby even more efficiently combating competing (micro) organisms.

### General stress response

As the natural habitats of bacteria are constantly subjected to deleterious and fluctuating conditions that can be harmful, bacteria all have evolved their abilities to sense and respond to these environmental changes. RpoS, RelA, universal stress protein A, starvation stringent protein (SspB) and phage shock proteins (PspA-E) that are involved in controlling carbon and nutrient starvation [8] are present in *P. flavipulchra* JG1. Furthermore, the genome of JG1 also encodes for a large number of proteins involved in oxidative stress and metal homeostasis.

Besides three antioxidative proteins of the AhpC/Tsa family, including catalase, superoxide dismutase and an alkyl hydroperoxide reductase, JG1 has two proteins of the AhpF/TR family identified as AhpF and thioredoxin reductase [46]. These antioxidant enzymes protect JG1 against peroxide derived DNA damage as well as oxidative membrane or lipids destruction. Key regulators of the oxidative stress response are also present (such as SoxR). Unlike *P. tunicata* D2 and *P. haloplanktis* TAC125 [13], the ubiquitous molybdopterin metabolism might be present, since dinucleotide-utilizing enzymes involved in molybdopterin biosynthesis can be found in JG1. However, genes coding for enzymes using cofactors, such as xanthine oxidase, biotin sulfoxide reductase [47], have not been detected. Five putative dioxygenases (FaGL2063, FaGL3237, FaGL2992, FaGL1118 and FaGL4206) might also help JG1 to protect its metabolism against oxidative stress.

Several genes involved in heavy metal detoxification were discovered in the genome of JG1, including periplasmic divalent cation tolerance protein (CutA) and copper homeostasis protein (CutC) [48]. Experiments proved that intracellular copper accumulation in *E. coli* could increase without *cutA*[49]. Moreover, *cutA* affects not only copper tolerance but also tolerance levels to zinc, nickel, cobalt and cadmium.

### Motility and secretion

**Figure 5:**
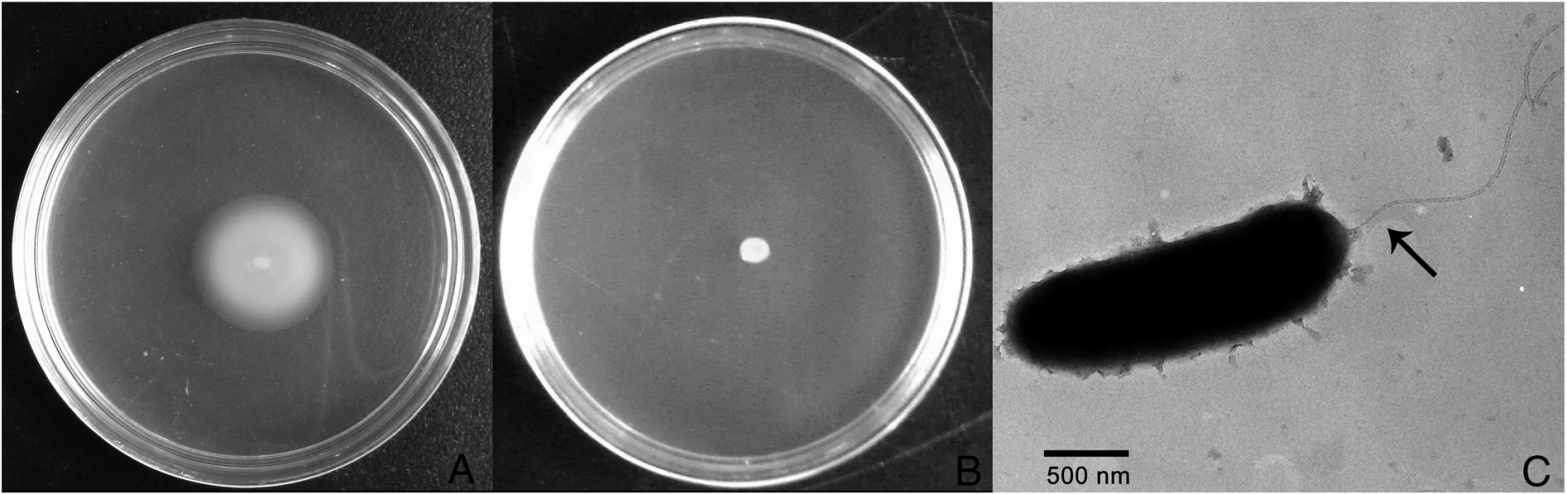
**Swimming and swarming motility and transmission electron microscopy of *P. flavipulchra *JG1. (A)** Distinct motility halo observed in the swimming plate; **(B)** No swarming ability observed in the swarming plate. **(C)** Transmission electron microscopy of *P. flavipulchra* JG1. The arrow indicated the polar flagellum.

Flagellum formation is an important response to environmental stress. The swimming motility of *P. flavipulchra* JG1 observed by swimming plate method showed a distinct motility halo (Figure 5A), while the swarming ability was not detected (Figure 5B). Genes for synthesis of flagellum are present in *P. flavipulchra* JG1, and the resulting polar flagellum is visible under the transmission electron microscope (Figure 5C). Thirty three genes are involved in flagellar assembly in the *P. flavipulchra* JG1 genome. The class III flagellar operons which are controlled negatively by *flgM* are present [50]. FlgM is an inhibitory factor to sense the integrity of the bacterial flagellar structure and regulate flagellar synthesis by binding the transcription factor σ^28^[51]. However, the transcriptional activators FlhC and FlhD protein [52] were missing. FlhD alone also regulates the cell division rate, limiting cell division of bacterial communities entering the stationary phase, as shown for *E. coli*[53]. The deletion of FlhD may help JG1 to maintain a stable division rate to more quickly respond to environmental changes thus giving competitive advantages.

*P. flavipulchra* JG1 possesses three gene clusters for the biosynthesis of type IV pili including PilM/N/O/P/Q and PilF which interact to allow pilus assembly to occur, and also PilA, which is the most important component of type IV pili [54]. Pili mediate attachment to both living and artificial surfaces and are involved in bacteriophage adsorption, DNA uptake, biofilm initiation and development, and twitching motility [55]. One of the pili gene clusters and its upstream region is highly conserved in *P. tunicata*, *P. haloplanktis* and *Pseudoalteromonas* sp. TW-7 and SM9913 contains homologs to the two component response regulator system *alg*Z/*alg*R, which is involved in the regulation of alginate synthesis and pili-mediated, twitching motility in *Pseudomonas aeruginosa*[56]. The alginate biosynthesis cluster is absent in JG1 as in *P. tunicata*, therefore, this regulatory system may only play a role in the expression of the pili cluster. With these appendages involved in motility, JG1 might be able to rapidly respond to environmental stresses and exceed other microorganisms.

There are several secretion systems functional in JG1, such as type I, II and VI secretion system, as well as the TAT and Sec-SRP export systems. In contrast, type III secretion system is absent. There are 15 genes involved in the type VI secretion system (T6SS). Previous studies indicated that many of these T6SS-containing bacteria are known pathogens and T6SS have been experimentally shown to play a role in virulence in several cases [57]. However, recent studies suggest that T6SS may limit bacterial replication or virulence and instead be used for intraspecies microbial cooperation, such as mediating antagonistic interactions between bacteria [58]. T6SS may be one of antibacterial mechanisms of JG1 that allows the bacterium inhibiting other microorganisms and affecting bacterial-host interactions.

### Drugs resistance and transport

Microorganisms have developed various ways to resist the toxic effects of antibiotics and other drugs [59,60]. JG1 was shown excellent resistant activities to several common antibiotics such as penicillin, kanamycin, cephalosporin, tetracycline and chloramphenicol. One of the mechanisms may be enzymes that inactivate antibiotics by hydrolysis or the formation of inactive derivatives [61]. Penicillin metabolism in JG1 could be catalyzed by penicillin amidase yielding 6-aminopenicillanic acid, and by beta-lactamase (penicillinase) to give penicilloic acid. The penicillin amidase can also be found in TAC125, but not in other compared *Pseudoalteromonas* strains. JG1 also possesses D-amino-acid oxidase to convert cephalosporin C into (7R)-7-(5-carboxy-5-oxopentanoyl)-aminocephalosporinate, consequently disarming this bactericide.

A second mechanism of antibiotic resistance is the inhibition of drug entry into the cell. The low permeability of the outer membrane of gram-negative bacteria could reduce drug diffusion across the cell envelope [62]. However, these barriers cannot entirely prevent all drugs from exerting their toxic action once they have entered the cell. The active efflux of drugs is thus essential to ensure the survival of the cell [63,64]. JG1 possesses many genes involved in defense mechanisms due to ABC-type antimicrobial peptide transport system (34 genes), ABC-type multidrug transport system (16 genes), cation/multidrug efflux pump (12 genes) and Na^+^-driven multidrug efflux pump (7 genes). The ABC-type transport systems have genes encoding ATPase and permease components. These multidrug transporters recognize lipophilic drugs by their physic-chemical properties that allow them to intercalate into the lipid bilayer, and transport these agents from the lipid bilayer to the exterior. The five small molecular compounds with antibacterial activity produced by JG1 were all lipophilic substances and multidrug transporters could reduce the intracellular accumulation of these compounds and protect the bacterium from the toxic effect of these bacteriostats. The transporters mediate the excretion of specific antibiotics in *Streptomyces* strains also dedicated to ensure self-resistance to the antibiotics that they produce [65].

## Conclusions

The genome of *P. flavipulchra* JG1 unveils significant genetic advantages against other microorganisms, encoding antimicrobial agents as well as abilities to adapt to various adverse environments. The antibacterial protein PfaP not only catalytically produces hydrogen peroxide as a bacteriostat but likely also participates in the biosynthesis of small molecular antibacterial compound (6-bromoindolyl-3-acetic acid). Both the macromolecule and small molecules contribute to the antibacterial activities of JG1. Besides these already identified chemical structures produced by strain JG1, a large number of peptide-based secondary metabolites encoded in the genome still awaits discovery. The identification of various antimicrobial enzymes enriches the antagonistic mechanisms of *P. flavipulchra* JG1 and could serve as therapeutic strategies against aquaculture pathogens. Furthermore, JG1 also evolves a range of mechanisms adapting the adverse marine environment or multidrug rearing conditions. The analysis of the genome of *P. flavipulchra* JG1 presented here provides a better understanding of its competitive properties and also an extensive application prospect.

## Methods

### Bacterial growth and DNA extraction

*P. flavipulchra* JG1 was isolated from rearing water of healthy turbot (*Scophthalmus maximus*) in Qingdao, China and was routinely grown on marine agar 2216 (MA; Difco) at 28°C. Genomic DNA was extracted from 5 ml overnight culture by standard methods [66]. Genomic DNA was quantified on 1% agarose gel stained with ethidium bromide and assessed spectrophotometrically.

The biosensors *Chromobacterium violaceum* CV026 and VIR24 were used to detect the short-chain (C4-C8) and long-chain (C8-C14) acyl homoserine lactones (AHLs), respectively. Both of them were grown on Luria–Bertani (LB) agar at 28°C.

### Genome sequencing, annotation and analysis

The genome sequence of *P. flavipulchra* JG1 was determined using the Illumina HiSeq2000 with a 500 bp paired-end library and achieved about 600 Mb data with 111.9-fold coverage. The reads were assembled using SOAPdenovo assembler software [67] subsequently. A total of 122 contigs ranging from 128 bp to 264 447 bp (the N50 and N90 contig sizes were 107 608 bp and 34 132 bp, respectively) were obtained and combined into 61 scaffolds ranging from 500 bp to 879 239 bp (the N50 and N90 contig sizes were 338 061 bp and 74 731 bp, respectively). Putative protein-encoding genes were identified with GLIMMER [68], transposons were predicted with Repeat Masker and Repeat Protein Masker [69], and tandem repeat sequences were identified through Tandem Repeat Finder [70]. Annotation was performed with BLASTALL 2.2.21 [71] searching against protein databases KEGG (Kyoto encyclopedia of genes and genomes; http://www.genome.jp/kegg/) [72], COG ( http://www.ncbi.nlm.nih.gov/COG/) [73], SwissProt and TrEMBL ( http://www.uniprot.org/) and NR (NCBI non-redundant database; http://www.ncbi.nlm.nih.gov/RefSeq/) [74]. The criteria used to assign function to a CDS were a minimum cutoff of 30% identity and at least four best hits among the COG, KEGG, NR, SwissProt or TrEMBL protein databases. Phylogenetic analysis was performed by alignment of sequences using Clustal W [75] and neighbor-joining trees were generated with 1 000 bootstraps. The prediction of signal peptides (SP) was performed using SignalP v 4.1 [76]. The conserved domains were predicted using Conserved Domain Database of NCBI [77].

### Nucleotide sequence accession numbers

This Whole Genome Shotgun project has been deposited at DDBJ/EMBL/GenBank under the accession AJMP00000000. The version described in this paper is the first version, AJMP01000000.

### Comparative genomics

According to the result of phylogenetic analysis, the genome sequences of *P. tunicata* D2 and *P. haloplanktis* TAC125 were retrieved from NCBI. Proteins from *P. flavipulchra* JG1 were compared with those of D2 and TAC125, using BLASTP with an E-value cutoff of 1e-5. Orthologous proteins are defined as reciprocal best hit proteins with a minimum 40% identity and 70% of the length of the query protein, calculated by the BLAST algorithm. Proteins without orthologs are considered to be specific proteins. The COG function category was analyzed by searching all predicted proteins against the COG database on the basis of the BLASTP.

### Catalase effect on the antibacterial activity

An 8 mm disc loaded with 0.1 mg, 0.2 mg and 0.5 mg of catalase in 10 μl distilled water was used and placed neighboring to the circular wells loaded with 20 μl extracellular proteins of *P. flavipulchra* JG1, respectively [14]. The catalase inhibition of antibacterial effect was observed by the eclipse of inhibition areas.

### AHLs degradation bioassay

C6 to C14-HSL and 3-oxo-C6 to 3-oxo-C14-HSL were used for evaluating the AHL degradation activity of *P. flavipulchra* JG1. Briefly, extra- and intra-cellular products of strain JG1 were mixed with different acyl chains of AHLs, the final concentrations of these AHLs were 1 μM for C10-HSL, C12-HSL and C14-HSL, and 0.1 μM for the rest AHLs. The mixtures were incubated at 28°C for 24 hours and the residual AHLs were detected by *Chromobacterium violaceum* CV026 and VIR24 plate assay [78].

### Electron microscopy and motility assay

An overnight culture of strain JG1 was negatively stained with 1% phosphotungstic acid (pH 7.4) on a Formvar carbon-coated grid and observed with a transmission electron microscope (TEM-1200EX, Japan) [79]. Swimming and swarming motilities were evaluated by point inoculating JG1 on MA plates containing 0.3% and 0.5% agar, respectively. The plates were analyzed after incubation at 28°C for about 24 h. The experiment was performed in triplicate.

### Chitinase activity and antibiotic resistance

The chitinase activity of *P. flavipulchra* JG1 were observed using chitin agar following the method described by Hsu *et al.*[80]. Resistance to antibiotics of strain JG1 was investigated by the agar diffusion method using the filter discs containing different antibiotics. Briefly, 100 μl aliquots of overnight broth culture of JG1 were spread onto the surface of MA plates, and different antibiotic discs were placed on the target plates, respectively, which were then incubated at 28°C for 24 h. Inhibition zones of the antibiotics-containing discs were observed.

## Competing interests

The authors declare that they have no competing interests.

## Authors’ contributions

X-HZ and XS designed and oversighted the study. MY and KT performed the laboratory work, analyzed the data and drafted the manuscript, JL and TAMG analyzed the data. All authors read and approved the final manuscript.

## Supplementary Material

Additional file 1: Figure S1 Gene percentages assigned to all the COG categories of the orthologous and specific genes in *P. flavipulchra* JG1, *P. tunicata* D2 and *P. haloplanktis* TAC125. (A-C) Abundances of specific genes in *P. flavipulchra* JG1, *P. tunicata* D2 and *P. haloplanktis* TAC125 assigned to the COG categories. (D) Orthologous genes among these three genomes assigned to the COG categories. COG functional categories are described in Figure 2. Click here for file

Additional file 2: Table S1 Genes within the gene clusters involved in secondary metabolites synthesis. Click here for file
