# Supplementary figures and images for: Genome analysis of Pseudoalteromonas flavipulchra JG1 reveals various survival advantages in marine environment
Source: BMC Genomics. 2013 Oct 16;14:707. doi: 10.1186/1471-2164-14-707 (PMC3853003; doi:10.1186/1471-2164-14-707)

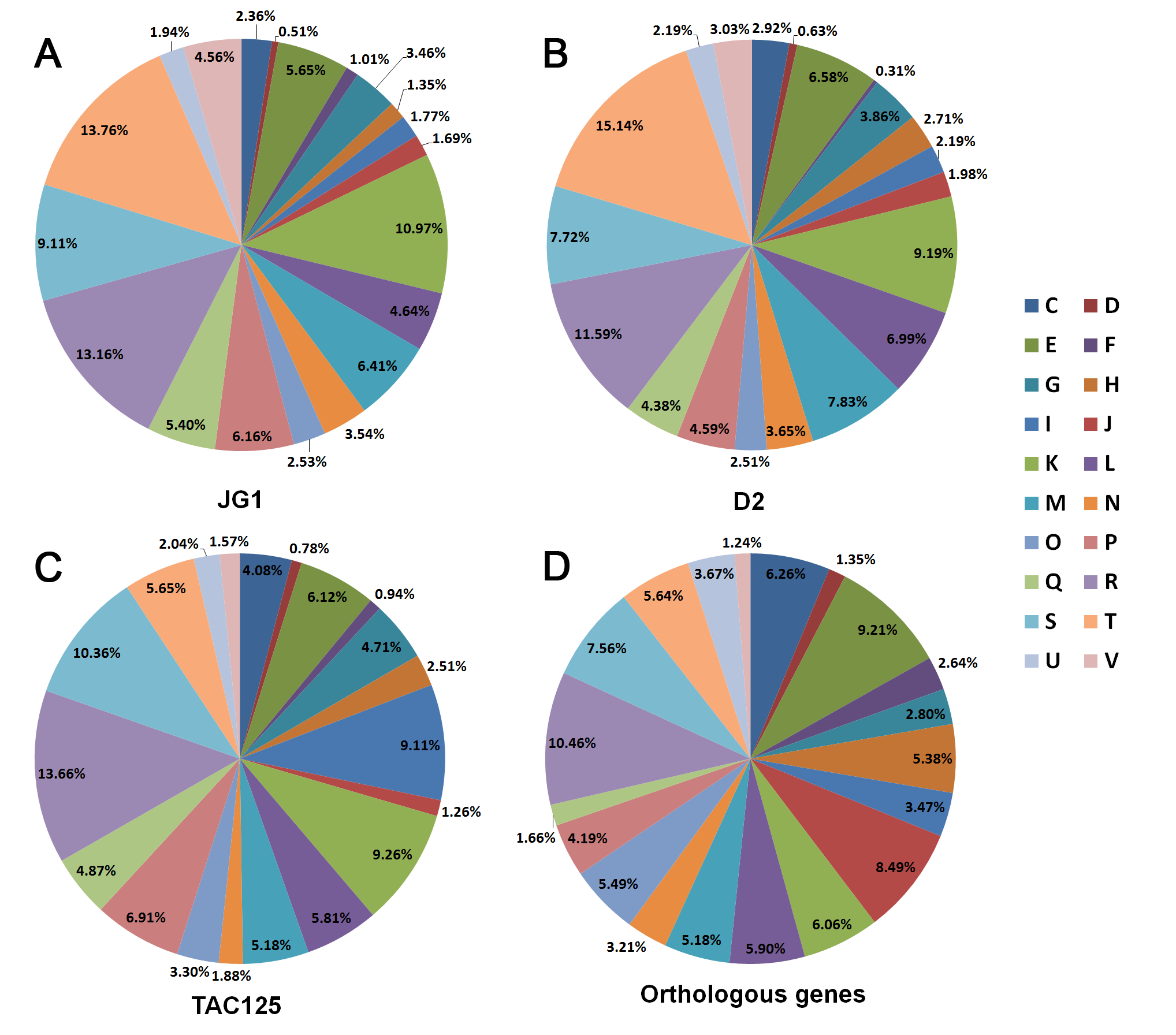

Supplement: Additional file 1: Figure S1 — Gene percentages assigned to all the COG categories of the orthologous and specific genes in P. flavipulchra JG1, P. tunicata D2 and P. haloplanktis TAC125. (A-C) Abundances of specific genes in P. flavipulchra JG1, P. tunicata D2 and P. haloplanktis TAC125 assigned to the COG categories. (D) Orthologous genes among these three genomes assigned to the COG categories. COG functional categories are described in Figure 2. [file 1471-2164-14-707-S1.tiff]
