# Supplementary material for: Genome analysis of Pseudoalteromonas flavipulchra JG1 reveals various survival advantages in marine environment
Source: BMC Genomics. 2013 Oct 16;14:707. doi: 10.1186/1471-2164-14-707 (PMC3853003; doi:10.1186/1471-2164-14-707)
Supplement: Additional file 2: Table S1 — Genes within the gene clusters involved in secondary metabolites synthesis. [file 1471-2164-14-707-S2.pdf]

Table S1 Genes within the gene clusters involved in secondary metabolites synthesis

Locus	Proteins size	Gene name	Discription
**bacteriocin-type gene cluster 1**			
FaGL2831	487	traK	conjugation protein
FaGL2832	37	-	hypothetical protein
FaGL2833	232	djlA	DnaJ like chaperone protein
FaGL2834	292	PHYH	phytanoyl-CoA hydroxylase
FaGL2835	367	-	hypothetical protein
FaGL2836	184	cobO	cob(I)alamin adenosyltransferase
FaGL2837	781	acsF	magnesium-protoporphyrin IX monomethyl ester (oxidative) cyclase
FaGL2838	41	-	putative transposase
FaGL2840	67	-	putative transposase
FaGL2841	441	hisS	histidyl-tRNA synthetase
**bacteriocin-type gene cluster 2**			
FaGL3672	419	-	hypothetical protein
FaGL3673	334	-	hypothetical protein
FaGL3674	124	-	hypothetical protein
FaGL3675	321	dusC	tRNA-dihydrouridine synthase C
FaGL3676	339	-	hypothetical protein
FaGL3677	278	-	hypothetical protein
FaGL3678	249	-	hypothetical protein
FaGL3680	181	apt	adenine phosphoribosyltransferase
FaGL3681	775	dnaX	DNA polymerase III subunit gamma/tau
FaGL3682	108	-	hypothetical protein
**bacteriocin-type gene cluster 3**			
FaGL4702	929	hisA	phosphoribosylformimino-5-aminoimidazole carboxamide ribotide isomerase
FaGL4703	302	pleD	two-component system, cell cycle response regulator
FaGL4704	330	oruR	ornithine utilization regulator
FaGL4705	149	-	hypothetical protein
FaGL4706	276	-	hypothetical protein, belongs to the UPF0276 family
FaGL4707	266	-	hypothetical protein
FaGL4708	37	-	hypothetical protein
FaGL4710	621	proA	alkalin serine protease
FaGL4711	280	-	AraC family transcriptional regulator

FaGL4712	701	fhuA	iron complex outermembrane recepter protein
**bacteriocin-type gene cluster 4**			
FaGL4805	1083	tri	tricorn protease
FaGL4806	293	-	hypothetical protein
FaGL4807	164	-	hypothetical protein
FaGL4808	775	-	hypothetical protein
FaGL4809	316	-	hypothetical protein, belongs to the UPF0276 family
FaGL4810	291	-	hypothetical protein
FaGL4811	648	-	non-specific serine/threonine protein kinase
FaGL4812	373	-	hypothetical protein
FaGL4813	121	-	hypothetical protein
FaGL4814	634	dppV	Dipeptidyl-peptidase 5
**lantipeptide biosynthesis gene cluster (FaGL4338-FaGL4382)**			
**key genes**			
FaGL4346	239	araC	AraC family transcriptional regulator
FaGL4347	888	hisA	phosphoribosylformimino-5-aminoimidazole carboxamide ribotide isomerase
FaGL4348	501	-	tryptophan halogenase
FaGL 4349	421	fucP	MFS transporter, FHS family, L-fucose permease
FaGL 4350	291	glk	glucokinase
FaGL 4351	842	-	hexosaminidase
FaGL 4354	533	arsO	putative flavoprotein involved in K^+ transport
FaGL4355	1042	epiB	epidermin biosynthesis protein
FaGL4356	386	spaC	subtilin biosynthesis protein
FaGL4358	169	cvaB	ATP-binding cassette, subfamily B
FaGL4362	307	panE	2-dehydropantoate 2-reductase
FaGL4364	411	carB	carbamoyl-phosphate synthase large subunit
FaGL4368	412	iaaL	indoleacetate---lysine synthetase
FaGL4369	477	fadD	long-chain acyl-CoA synthetase
FaGL4370	429	fabF	3-oxoacyl-[acyl-carrier-protein] synthase II
FaGL4371	365	fabF	3-oxoacyl-[acyl-carrier-protein] synthase I
FaGL4373	238	fabG	3-oxoacyl-[acyl-carrier protein] reductase
FaGL4376	264	-	glucose-6-phosphate 1-epimerase
FaGL4377	251	acpT	4'-phosphopantetheinyl transferase
FaGL4380	231	-	putative ABC transport system ATP-binding protein
FaGL4382	255	algR	two-component system, LytT family, response regulator
**type I polyketide/non-ribosomal peptide (PKS/NRPS) hybrid cluster 1 (FaGL0407-FaGL0461)**			
**key genes**			

FaGL0407	217	yesM	two-component system, LytT family, sensor kinase
FaGL0408	518	ybaR	sulfate permease, SulP family
FaGL0410	152	ybaO	Lrp/AsnC family transcriptional regulator
FaGL0411	213	gloB	hydroxyacylglutathione hydrolase
FaGL0412	589	-	hypothetical protein
FaGL0413	290	-	short chain dehydrogenase
FaGL0414	119	rplT	50S ribosomal protein L20
FaGL0415	77	rpmI	50S ribosomal subunit protein A
FaGL0416	187	infC	translation initiation factor IF-3
FaGL0417	636	thrS	threonyl-tRNA synthetase
FaGL0421	614	-	ATP-binding cassette, subfamily B
FaGL0422	200	rutR	TetR/AcrR family transcriptional regulator
FaGL0423	374	acrA	AcrA/E family efflux transporter MFP subunit
FaGL0424	1039	-	hydrophobic/amphiphilic exporter-1 (mainly G^- bacteria), HAE1 family
FaGL0425	584	-	fatty-acyl-CoA synthase
FaGL0426	480	katE	catalase
FaGL0428	445	ntrY	two-component system, NtrC family, nitrogen regulation sensor histidine kinase NtrY
FaGL0429	439	-	two-component system, NtrC family, response regulator
FaGL0430	406	-	putative ABC transport system permease protein
FaGL0431	453	-	putative ABC transport system permease protein
FaGL0432	231	-	putative ABC transport system ATP-binding protein
FaGL0433	422	-	HlyD family secretion protein
FaGL0434	409	lolE	lipoprotein releasing system transmembrane protein
FaGL0435	229	lolD	lipoprotein-releasing system ATP-binding protein
FaGL0436	399	lolC	lipoprotein-releasing system permease protein
FaGL0437	159	lrp	Lrp/AsnC family transcriptional regulator, leucine-responsive regulatory protein
FaGL0441	434	dapE	succinyl-diaminopimelate desuccinylase
FaGL0442	3113	-	beta-ketoacyl synthase
FaGL0443	551	crtH	prolycopene isomerase
FaGL0444	178	fldB	flavodoxin II
FaGL0445	554	crtH	prolycopene isomerase
FaGL0446	318	-	lathosterol oxidase
FaGL0447	463	norM	multidrug resistance protein, MATE family
FaGL0448	263	-	oleoyl-[acyl-carrier-protein] hydrolase
FaGL0449	434	tlyC	putative hemolysin

FaGL0452	100	-	7,8-dihydropterin-6-yl-methyl-4-(beta-D-ribofuranosyl)aminobenzene 5'-phosphate synthase
FaGL0453	430	msrC	peptide methionine sulfoxide reductase
FaGL0455	504	pepA	leucyl aminopeptidase
FaGL0456	216	-	carboxylase
FaGL0457	312	-	allophanate hydrolase
FaGL0460	220	pcp	pyroglutamyl-peptidase
FaGL0461	533	asnB	asparagine synthase (glutamine-hydrolysing)
**type I PKS/NRPS hybrid cluster 2 (FaGL4470-FaGL4515)**			
**key genes**			
FaGL4480	183	rpoE	RNA polymerase sigma-70 factor, ECF subfamily
FaGL4487	144	zur	Fur family transcriptional regulator, zinc uptake regulator
FaGL4488	756	cheBR	two-component system, chemotaxis family, CheB/CheR fusion protein
FaGL4489	586	ggt	gamma-glutamyltranspeptidase
FaGL4491	3326	-	amino acid adenylation domain-containing protein
FaGL4492	3275	lgrD	linear gramicidin synthetase subunit D
FaGL4493	4022	lgrD	linear gramicidin synthetase subunit D
FaGL4495	448	-	D-alanyl-D-alanine carboxypeptidase
FaGL4498	527	pvdE	putative ATP-binding cassette transporter
FaGL4505	448	accC	acetyl-CoA carboxylase, biotin carboxylase subunit
FaGL4506	154	accB	acetyl-CoA carboxylase biotin carboxyl carrier protein
FaGL4515	382	argE	acetylornithine deacetylase
**type I PKS/NRPS hybrid cluster 3 (FaGL1419-FaGL1460)**			
**key genes**			
FaGL1419	3426	lgrD	linear gramicidin synthetase subunit D
FaGL1420	3206	lgrD	linear gramicidin synthetase subunit D
FaGL1421	573	pvdE	putative ATP-binding cassette transporter
FaGL1423	447	-	HlyD family secretion protein
FaGL1424	237	-	putative ABC transport system ATP-binding protein
FaGL1427	460	-	two-component system, NtrC family, response regulator
FaGL1428	396	ntrY	two-component system, NtrC family, nitrogen regulation sensor histidine kinase
FaGL1433	434	pvdA	L-ornithine N5-oxygenase
FaGL1434	732	-	iron complex outermembrane recepter protein
FaGL1435	2477	-	aspartate racemase
FaGL1436	1127	-	aspartate racemase
FaGL1437	1512	-	glutamate-1-semialdehyde 2,1-aminomutase

FaGL1438	1818	paeJ	polyketide synthase
FaGL1439	246	-	oleoyl-[acyl-carrier-protein] hydrolase
FaGL1441	538	pvdE	pyoverdine synthetase E
FaGL1442	372	dapE	succinyl-diaminopimelate desuccinylase
FaGL1444	210	iucB	acetyl CoA:N6-hydroxylysine acetyl transferase
FaGL1445	314	yeaP	diguanylate cyclase
FaGL1453	306	dgcB	diguanylate cyclase
FaGL1454	379	proB/	glutamate 5-kinase
**type I PKS/NRPS hybrid cluster 4 (FaGL2543-FaGL2561)**			
**key genes**			
FaGL2544	592	-	ATP-binding cassette, subfamily C
FaGL2548	411	sufS	cysteine desulfurase / selenocysteine lyase
FaGL2550	465	glnG	two-component system, NtrC family, nitrogen regulation response regulator
FaGL2551	360	glnL	two-component system, NtrC family, nitrogen regulation sensor histidine kinase
FaGL2555	576	pvdE	putative ATP-binding cassette transporter
FaGL2556	1045	dhbF	nonribosomal peptide synthetase
FaGL2557	1801	ppsE	phenolpthiocerol synthesis type-I polyketide synthase E
FaGL2558	1097	-	amino acid adenylation domain-containing protein
FaGL2559	1136	dhbF	nonribosomal peptide synthetase
FaGL2560	256	ectD	ectoine hydroxylase
FaGL2561	2207	-	amino acid adenylation
**NRPS gene cluster 1 (FaGL3492-FaGL3512)**			
**key genes**			
FaGL3492	1517	-	photinus-luciferin 4-monooxygenase (ATP-hydrolysing)
FaGL3493	251	-	oleoyl-[acyl-carrier-protein] hydrolase
FaGL3495	243	fabG	3-oxoacyl-[acyl-carrier protein] reductase
FaGL3496	132	fabZ	3-hydroxyacyl-[acyl-carrier-protein] dehydratase
FaGL3497	371	-	ABC-2 type transport system permease protein
FaGL3498	207	-	ABC-2 type transport system ATP-binding protein
FaGL3499	727	fabF	3-oxoacyl-[acyl-carrier-protein] synthase II
FaGL3500	498	-	4-coumarate--CoA ligase
FaGL3501	112	acpP	acyl carrier protein
FaGL3502	539	hutH	histidine ammonia-lyase
FaGL3504	197	yvfR	ABC-2 type transport system ATP-binding protein
FaGL3506	662	toxR	cholera toxin transcriptional activator
FaGL3508	817	fadE	acyl-CoA dehydrogenase

FaGL3510	374	pdxB	erythronate-4-phosphate dehydrogenase
FaGL3511	338	asd	aspartate-semialdehyde dehydrogenase
**NRPS gene cluster 2 (FaGL4138-FaGL4183)**			
**key genes**			
FaGL4154	279	rexA	oligosaccharide reducing-end xylanase
FaGL4155	850	-	serine/threonine protein kinase
FaGL4161	297	ydhF	oxidoreductase
FaGL4162	405	purT	phosphoribosylglycinamide formyltransferase 2
FaGL4163	1660	dhbF	nonribosomal peptide synthetase
FaGL4164	2103	-	nonribosomal peptide synthetase
FaGL4165	1370	-	photinus-luciferin 4-monooxygenase (ATP-hydrolysing)
FaGL4169	318	natA_1	ABC-2 type transport system ATP-binding protein
FaGL4171	293	-	ABC transporter ATP-binding protein
FaGL4172	225	-	nitroreductase
FaGL4173	348	mdtA	putative multidrug efflux transporter
FaGL4174	1077	-	hydrophobic/amphiphilic exporter-1 (mainly G- bacteria), HAE1 family
FaGL4175	101	arsR	ArsR family transcriptional regulator
FaGL4176	114	gloB	hydroxyacylglutathione hydrolase
FaGL4179	252	-	putative PAS/PAC sensor protein
FaGL4182	409	mscS	small conductance mechanosensitive channel
FaGL4183	306	-	acyltransferase
**NRPS gene cluster 3 (FaGL1775-FaGL1792)**			
**key genes**			
FaGL1775	2241	mps1	glycopeptidolipid biosynthesis protein
FaGL1776	412	-	dehydrogenase
FaGL1779	411	mdtH	MFS transporter, DHA1 family, multidrug resistance protein
FaGL1780	171	pagL	lipid A 3-O-deacylase
FaGL1781	248	-	polar amino acid transport system substrate-binding protein
FaGL1782	794	ygjK	putative isomerase
FaGL1783	400	adh	alcohol dehydrogenase
FaGL1784	156	rpoE	RNA polymerase sigma-70 factor, ECF subfamily
FaGL1785	321	fecR	transmembrane sensor
FaGL1786	909	tbpA	hemoglobin/transferrin/lactoferrin receptor protein
FaGL1787	211	hemO	heme oxygenase
FaGL1789	674	mcp	methyl-accepting chemotaxis protein
FaGL1792	324	-	AraC family transcriptional regulator
